# Uncovering the origins of a niche

**DOI:** 10.7554/eLife.05041

**Published:** 2014-11-11

**Authors:** Jeff M Bernitz, Kateri A Moore

**Affiliations:** 1 **Jeff M Bernitz** is in the Department of Developmental and Regenerative Biology, Black Family Stem Cell Institute, Graduate School of Biomedical Sciences, Icahn School of Medicine at Mount Sinai, New York, United States; 2 **Kateri A Moore** is in the Department of Developmental and Regenerative Biology, Black Family Stem Cell Institute, Icahn School of Medicine at Mount Sinai, New York, United Stateskateri.moore@mssm.edu

**Keywords:** mesenchymal stem cells, hematopoietic stem cells, stem cell niche, bone marrow, neural crest, mouse

## Abstract

Multiple cell types that share a common origin cooperate to form a supportive niche for stem cells that give rise to blood and to the cells of the immune system.

**Related research article** Isern J, García-García A, Martín AM, Arranz L, Martín-Pérez D, Torroja C, Sánchez-Cabo F, Méndez-Ferrer S. 2014. The neural crest is a source of mesenchymal stem cells with specialized hematopoietic stem cell niche function. *eLife* **3**:e03696. doi: 10.7554/eLife.03696

**Image** Bone marrow sections from one-week-old mice reveal distinct cell types that form a stem cell niche

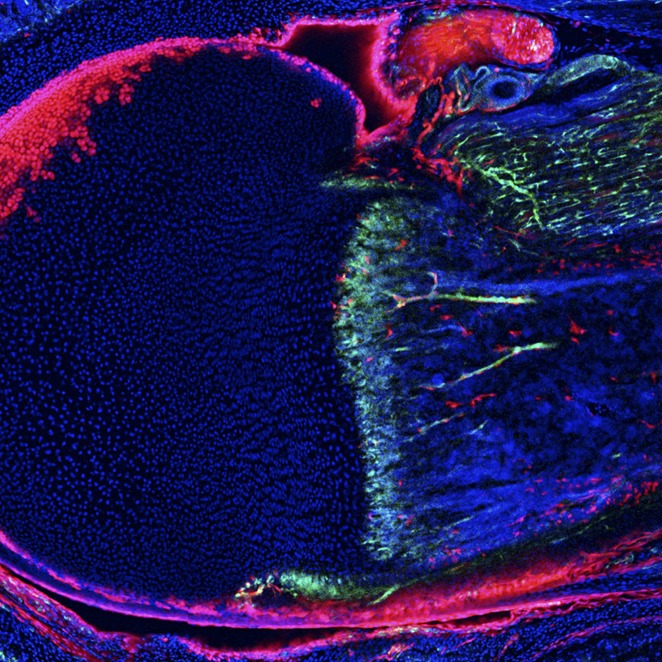

The formation of an embryo is a very well-ordered process. During the 6th day after fertilisation, a mouse embryo forms three distinct cell layers—called the endoderm, the mesoderm, and the ectoderm. Each layer gives rise to distinct tissues and organs within the body. The endoderm forms the lungs and the gastrointestinal tract (which includes the liver and pancreas); the mesoderm generates the kidneys, bones, blood, muscle and heart; and the ectoderm differentiates to form numerous tissues including the nervous system and skin.

The tissues of a developing embryo rapidly divide and contain so-called ‘progenitor cells’ that can generate the specialised cell types needed to make a body. After birth, the tissues lose their ability to proliferate in this manner. Instead limited populations of stem cells are set aside so that they can be used later in life to regenerate and repair tissues that become old or damaged. However, the regenerative potential of these stem cells is limited to the specific tissue or organ system within which they reside.

The stem cells that produce all the cells in the blood and the immune system are called haematopoietic stem cells (or HSCs for short). During embryonic development HSCs are found in different sites at different stages. In adults HSCs are found in the bone marrow. The bone marrow is also home to a second group of stem cells called mesenchymal stem cells (MSCs), which maintain and repair the skeleton by producing cells that make bone, cartilage and fat. In 2010 it was discovered, unexpectedly, that MSCs form part of the niche that maintains HSCs in the bone marrow (Méndez-Ferrer et al., 2010).

A stem cell niche is composed of cells and other physical components that work together to protect, instruct and nurture stem cells. Over the years many different cell types have been identified as components of the HSC niche. The majority of these cells originate from the mesoderm, and include various cells that make up and surround blood vessels and bone (Ding et al., 2012; Ding and Morrison, 2013; Greenbaum et al., 2013). Additionally, other studies have reported that ectodermal cells from the nervous system are important in supporting HSCs (Katayama et al., 2006; Yamazaki et al., 2011; Mendelson and Frenette, 2014). Given the diverse range of niche components, Simón Méndez-Ferrer and colleagues at the Centro Nacional de Investigaciones Cardiovasculares in Madrid set out to determine the developmental origins and function of some of these cells, both before and after birth, and in adult life.

Now in *eLife*, Méndez-Ferrer and co-workers—including Joan Isern as the first author—report that, in mice, developing bones contain two populations of MSCs (Isern et al., 2014). One population contributes to the formation of the skeleton during embryonic development, but quickly loses its stem cell potential after birth. The second population—which is defined by the expression of a gene called *nestin*—does not contribute to bone and cartilage formation during development, but becomes the only population with MSC activity after birth. Moreover, these Nestin-positive MSCs are the same cells that play a critical role in the HSC niche (Méndez-Ferrer et al., 2010).

Next, Isern et al. sought to determine the developmental origins of these two MSC populations. Using a wide range of genetic experiments, they were able to follow the location and tissue of origin of each of these cell types during the development of mouse embryos. Isern et al. show that the skeleton-forming MSCs are derived from mesoderm tissue (Figure 1). The Nestin-positive MSCs, however, are derived from the neural crest; this is a short-lived population of cells in the ectoderm that gives rise to cells of the peripheral nervous system, as well as several other non-neural cell types. Additionally, Nestin-positive cells were found to include both MSCs and the precursors of Schwann cells (cells that wrap around and support nerve fibres). Schwann cell precursors can be distinguished from MSCs because they do not express a protein called ‘platelet-derived growth factor receptor α’ (or PDGFRα), whereas MSCs do (Figure 1).

**Figure 1.**
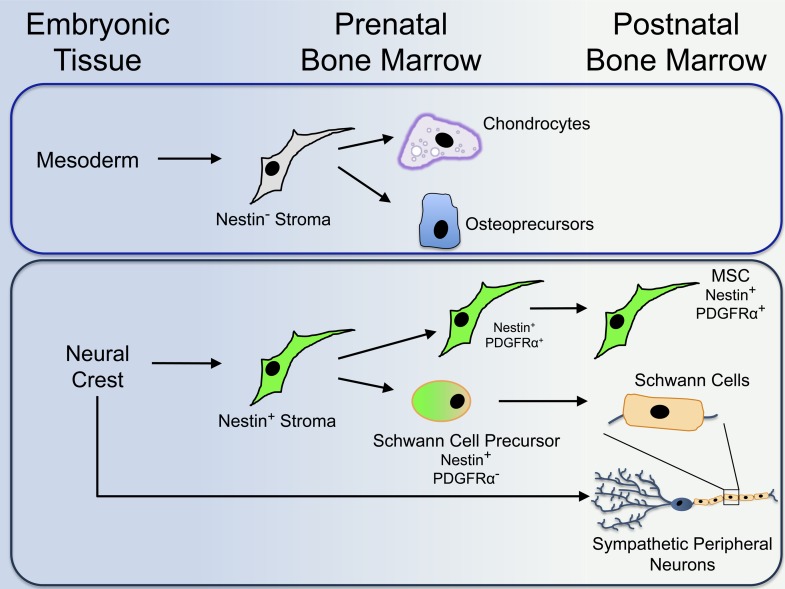
The protein Nestin segregates distinct populations of cells in the bone marrow that originate from different embryonic tissues during development. Isern et al. reveal that mesoderm-derived Nestin-negative (or Nestin^−^) bone marrow cells (or stroma) give rise to cells that contribute to the formation of cartilage (chondrocytes) and bone (osteoprecursors) in the foetus (top). Shortly after birth, Nestin-negative bone marrow cells lose their ability to generate further mature cells. The neural crest develops from the ectoderm. Nestin-positive (or Nestin^+^) cells derived from the neural crest give rise to Nestin-positive MSCs (mesenchymal stem cells). The Nestin-positive cells also give rise to Schwann cell precursors, which develop into mature Schwann cells in the bone marrow of newborn mice (bottom). Both populations of cells work together with neural crest-derived sympathetic peripheral neurons to establish a three-part niche for HSCs (haematopoietic stem cells). Unlike Nestin-negative bone marrow cells, Nestin-positive MSCs maintain the potential to develop into other types of cells after birth.

Finally, Isern et al. addressed the functional relevance of these populations of cells to the HSC niche. By deleting a gene critical to the migration of neural crest cells, they found that both Nestin-postive MSCs and Schwann cells fail to colonise the bone marrow. This, in turn, reduces the number of blood-forming progenitor cells that migrate to the bone marrow, which is a crucial step in the transition from foetal to adult blood formation. To confirm that these cells are important in establishing an HSC niche, Isern et al. also selectively deleted the Nestin-positive cells. This also prevented blood-forming progenitors from migrating to the bone marrow. Instead, the blood-forming progenitors were retained in the foetal liver where they are normally located prior to colonising the bone marrow.

Together, Isern et al.'s data show that Nestin-positive MSCs help establish a niche for HSCs in the bone marrow during development, and that multiple cell types derived from neural crest cells—Nestin-positive MSCs, Schwann cells (Yamazaki et al., 2011), and previously reported sympathetic nerve fibres (Katayama et al., 2006)—cooperate to form this niche. As putative MSC populations have also been reported in multiple other tissues—including skin, muscle and fat—it will be interesting to see if this three-part niche supports other stem cell populations throughout the body.
